# The Dominance of Severe Acute Respiratory Syndrome Coronavirus 2 B.1.617 and Its Sublineages and Associations with Mortality during the COVID-19 Pandemic in India between 2020 and 2021

**DOI:** 10.4269/ajtmh.21-0812

**Published:** 2021-11-17

**Authors:** Bakilapadavu Venkatraja, Gali Srilakshminarayana, Ballamoole Krishna Kumar

**Affiliations:** ^1^ Department of Economics, SDM Institute for Management Development, Mysuru, Karnataka, India;; ^2^ Department of Quantitative Methods, SDM Institute for Management Development, Mysuru, Karnataka, India;; ^3^ Nitte (Deemed to Be University), Division of Infectious Diseases, Nitte University Centre for Science Education and Research, Deralakatte, Mangalore, Karnataka, India

## Abstract

As the COVD-19 pandemic spreads, several new severe acute respiratory syndrome coronavirus 2 (SARS-COV-2) variants with various mutations across the genome have arisen, and they appear to be the greater risk to global public health. In this study, we have performed molecular characterization of SARS-COV-2 circulating in India between January 2020 and May 2021. Phylogenetic analysis of the SARS-COV-2 reported in the first and second waves of the outbreak showed the evolutionary hierarchy of SARS-COV-2 that was dispersed across the evolutionary tree of SARS-COV-2 with six major next strain clades: 19A (5.3%), 20A (29.9%), 20B (24.9%), 20I-Alpha, V1 (7.4%), 21A-Delta (17.2%), and 21B-Kappa (12.7%). Among the observed clades, 21A-Delta and 21B-Kappa belonging to the B.1.617 and its sublineages are the two notable clades that dominated approximately 78% of the total SARS-COV-2 genomes reported during April and May 2021. This study has also established a link between different SARS-COV-2 variants and risk of mortality during the COVID-19 epidemic using multivariable logistic regression model for patient-level data. The estimated model demonstrates that the risk of mortality of the COVID 19 patients infected by variant B.1.617 and/or its sublineages is much higher than the other preexisting SARS-COV-2 variants, especially among individuals over 45 years of age, regardless of gender. Considering the transmissibility of the B.1.617 and its sublineages and its potential impact to the public health, real-time analysis of COVID-19 cases coupled with stringent genomics surveillance are promising tools to develop and adapt stringent measures to contain and reduce the impact of SARS-COV-2.

## INTRODUCTION

COVID-19 is an emerging infectious disease caused by the severe acute respiratory syndrome coronavirus 2 (SARS-COV-2) that has posed an unprecedented challenge to public health. The first confirmed case of COVID-19 in India was reported in late January 2020, followed by a sudden surge in COVID-19 cases. According to available data on COVID-19 cases, the country has experienced two waves, with more than 28 million reverse transcriptase polymerase chain reaction (RT-PCR)-verified COVID-19 cases resulting in 329,100 deaths as of May 31, 2021 (https://www.COVID-19india.org). The first wave of COVID-19 began slowly in April 2020 and reached its peak in September 2020. India then encountered a devastating second wave of the pandemic, which proliferated around January 2021 and later increased rapidly before reaching the peak of more than 400,000 daily cases in early May 2021. It is significant to note that approximately 61% of the total COVID-19 cases in India and 53% of the total deaths were reported between February 2021 and May 2021. As the COVID-19 pandemic expands, SARS-COV-2 has undergone several mutations and genetic variations, resulting in the formation of different evolutionary groups used to classify viral strains into clades, lineages, and sublineages.^1^^–^^3^ In late 2020, new variants of SARS-COV-2, designated as “specific variants of interest (VOIs) and variants of concern (VOCs),” emerged with a greater risk to global public health.^4^^,^^5^ These variants possess multiple mutations across the genome, including several in the S protein and its receptor-binding domain.^6^^–^^8^ The resurgences in COVID-19 cases in the United Kingdom, South Africa, and Brazil coincides with a high prevalence of newly emerged VOCs such as B.1.1.7, B.1.351, and B.1.1.28; which has also disseminated to multiple countries worldwide.^9^^–^^12^ These three VOCs are likely to be associated with the increased transmission, higher infectivity and severity, and immune escape.^2^^,^^13^ Much like other countries, the second wave of COVID-19 in India, which started in January 2021, was also characterized by the emergence and/or circulation of a new variant named B.1.617.^8^^,^^14^ This newly emerged variant was characterized by having common signature mutations—D111D, G142D, L452R, E484Q, D614G, and P681R—in the spike protein, including within the receptor-binding domain (RBD).^14^ This newfound variant B.1.617 has seemingly replaced previous circulating SARS-COV-2 variants in India and even transmission across continents.^15^ Laboratory investigations have established the higher infectivity and increased pathological changes associated with the infection of newly emerged B.1.617 variants in laboratory animals. According to a hospital-based study, during the second wave (January–June 2021) the COVID-19 mortality rate was nearly 40% higher than the first wave (April–December 2020), leading to an unprecedented healthcare crisis in the country.^17^ The second wave began silently in India in early January 2021; positive cases rose steeply in April and peaked in early May. A few publications discuss the genetic changes and signature mutations in SARS-COV-2 strains circulating in different locations of India and have contributed to better understanding the genotypic of this virus.^4^^,^^8^^,^^12^ It has been hypothesized that this newly emerged variant could be partially responsible for India’s current surge in SARS-COV-2 infections and deaths, but there is no scientific evidence to justify this.^8^^,^^16^ Nonetheless, it is not certain whether this variant contributes to increased infectivity and clinical outcomes among infected patients. With this backdrop, we attempted to understand the association of genomic characteristics that may be responsible for the clinical outcomes of infected patients. For this purpose, we analyzed 5,740-genome datasets of SARS-COV-2 reported from India linked to patient metadata. The data were analyzed extensively using various standardized in silico tools and robust statistical models.

## METHODS

The analysis presented in this article is based on Indian SARS-COV-2 genomic sequences and their associated clinical metadata submitted by various contributors to the Global Initiative on Sharing Avian influenza Database (GISAID). A dataset of 15,740 whole-genome sequences of SARS-COV-2 virus reported from India between January 2020 and May 2021 was retrieved from the GISAID database based on the availability of patient metadata that includes clinical outcome and demographics. While retrieving the genome sequences, low–coverage sequences with > 5% Ns were excluded. All retrieved sequences were mapped to the reference genome of SARS-COV-2 deposited from Wuhan, China (GenBank Accession No. NC_045512.2). All 15,740 SARS-COV-2 sequences were subjected to comprehensive mutational profiling and phylogenomic analysis as described in the next section. Furthermore, all genome sequences were compared with metadata information such as patient mortality, recovery, patient age, and gender. In the data cleaning and filtering processes, several datasets were to be dropped from the analysis due to insufficient information, and finally the metadata for 840 SARS-COV-2 patients were found, providing complete information. The study thus used the data of 840 patients to understand the association of unique genetic characteristics of SARS-COV-2 with clinical outcome using inferential statistical analysis.

### Screening of mutations and phylogenetic analysis.

A phylogenetic dendrogram was constructed based on the complete genome sequences of 15740 Indian SARS-COV-2 strains using the Nextclade web-based tool (https://clades.nextstrain.org/). The selected sequences were uploaded to Nextclade webtool and aligned against genome sequence of SARS-COV-2 (Wuhan-Hu-1/2019) to assign Nextstrain clades and construct phylogeny trees of Indian SARS-COV-2 strains. The fasta files of SARS-COV-2 genomes were also subjected to Pangolin COVID-19 Lineage Assigner (https://pangolin.cog-uk.io/) for assigning Pango lineage based on the methodology described by Rambaut et al.^7^ Novel mutations in the genome of Indian SARS-COV-2 were analyzed with respect to Wuhan-Hu-1 (GenBank MN908947.3), which is regarded as the reference strain using webtool “coronapp” (http://giorgilab.unibo.it/coronannotator/) as per the method described by Mercatelli et al.^18^

### Statistical methods and tools.

The study employs relevant analytical methods to examine the research questions. One of the key questions is whether there exists any significant difference in the health status of COVID-19-infected patients based on their gender, and this was examined using χ^2^. We also examined whether there was a significant difference in the age distribution pattern of COVID-19 deceased individuals among the VOCs, and this was explored by applying Kruskal-Wallis test. Furthermore, a pairwise comparison test was performed to study the presence or absence of statistically significant differences in the mortality occurring from the infection of different VOCs. The study also deployed a correspondence analysis to explore whether the infection caused by the mutated virus over the period of interest has a different intensity of risk of mortality from the basal sequences.

While examining the primary objective of estimating the likelihood of the presence or absence of differences/bias in the mortality of the COVID-19-infected patients based on the VOCs, a multiple logistic regression model was built. The general form of the built model is presented in Equation (1).

Health Status=f(gender, age, variants of concern)  (1)

Health status is a binary and measures whether the infected person died or recovered from COVID-19. Gender denotes whether the infected individual is male or female. Age measures whether the infected person is young (< 45 years), adult (45–65 years), or old age (> 65 years). The VOCs are the genome sequences as categorized into Basel sequences: B.1, B.1.1, B.1.1.7, and B.1.617. Given this information, the model identifies the category of gender, age, and VOCs that have a significant impact on the probability of a patient dying from COVID-19 infection. The data on health status being categorical, application of logistic regression appeared to be the most appropriate statistical estimation.

We considered females aged < 45 years and infected with basal variants of SARS-COV-2 as the baseline or base category. This enables not only comparison of one category with another but also measurement of the size of change. From the early literature, it is evident that females, young age groups, and variants of early-period COVID-19 were associated with lower risk of mortality, and it is reasonable to consider them as the baseline category. Given the baseline category as specified, the final model estimated is as presented in Equation (2).

$$\text{log}\left(\frac{P\left(\text{deceased}\right)}{1-P\left(\text{deceased}\right)}\right)=\text{β0}\;\text{+}\;\text{β1DMale}\;\text{+}\;\text{β2DAdult}\;\text{+}\;\text{β3DOld}\;\text{+}\;\text{β4DB}\text{.1}\;\text{+}\;\text{β5DB}\text{.1}\text{.1}\;\text{+}\;\text{β6DB}\text{.1}\text{.1}\text{.7}\;\text{+}\;\text{β7DB}\text{.1}\text{.6}17$$ (2)

where, $\text{log}\left(\frac{P\left(\text{deceased}\right)}{1-P\left(\text{deceased}\right)}\right)$ is the response variable in the model and it measures the log odds of death of the patient against recovery,  P(deceased)  is the probability of the infected individual’s death, 1−P(deceased) denotes the probability of the recovery of the infected individual, β_0_ is the intercept of the model which measures the odds of demise of the individuals in the base category and β_1_, β_2_, β_3_, β_4_, β_5_, β_6_, and β_7_ are the coefficient values of their respective regressors.

## RESULTS AND DISCUSSION

### Mutation analysis.

The available 15,740 Indian SARS-COV-2 genomes were analyzed to identify the mutations accumulated in the viral genomes. There were more than 175 (reported in at least 1% genomes were included) mutations spread over different coordinates of Indian SARS-COV-2 isolates. Among those, mutations such as S: D614G, 5'UTR: 241; NSP3: F106F, and NSP12b: P314L were the most frequently occurring mutations and were found in more than 92% of genomes analyzed. The significant mutational landscape of Indian SARS-COV-2 follows the global trends where D614G was found to be more prevalent in the global pandemic and associated with mutations in nonstructural protein 3 (NSP3), RNA-dependent RNA polymerase (RdRp), and 5' UTR region.^19^ Clinical and in vitro experiments suggest that the accumulation of such unique mutations in the SARS-COV-2 genome may have an impact on transmission, disease severity, and vaccine development.^3^^,^^19^ Studies have also observed that SARS-COV-2 carrying D614G has obvious implications on the recovery of COVID-19 patients.^20^ Besides these dominant mutations, several sporadic and unique mutations were also found in the genomes analyzed. The top 15 unique nonsynonymous mutations frequently found in the Indian SARS-COV-2 genomes were analyzed in this study and are depicted in Supplemental Figure 1, which indicates the change in amino acid residues of their corresponding protein. The accumulation of mutations in the viral genome has also been shown to increase genetic variation, which served as the basis for determining the phylogenetic relationship of global SARS-COV-2 sequences, dividing them into multiple clusters/clades and lineages.^7^ Experts in the field predict that as the current pandemic continues, many new mutations may emerge alongside the genomic sequences, with clinical and pharmacological implications.^20^^,^^21^

### Phylogenetic analysis.

**Figure 1.**
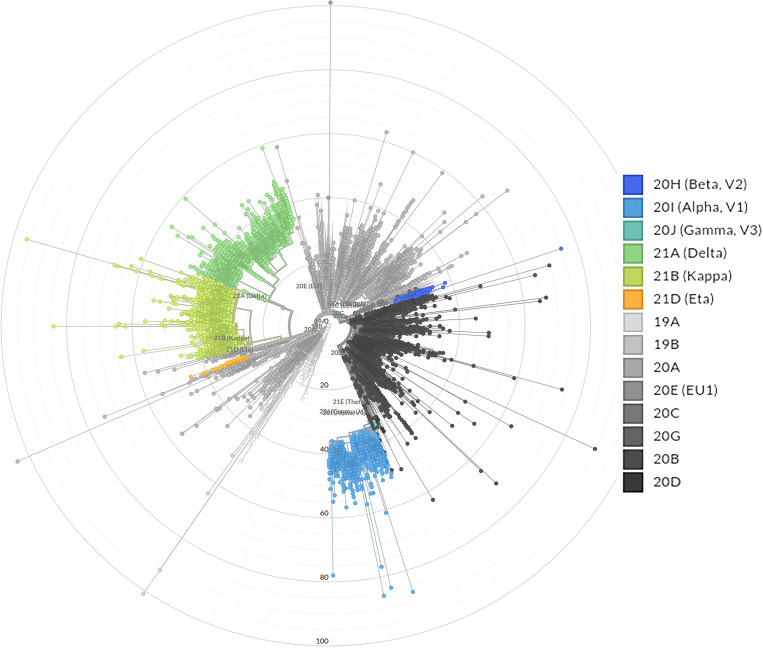
Phylogenetic tree showing the evolutionary relationship among various severe acute respiratory syndrome coronavirus 2 lineages circulating in India. This figure appears in color at www.ajtmh.org.

**Figure 2.**
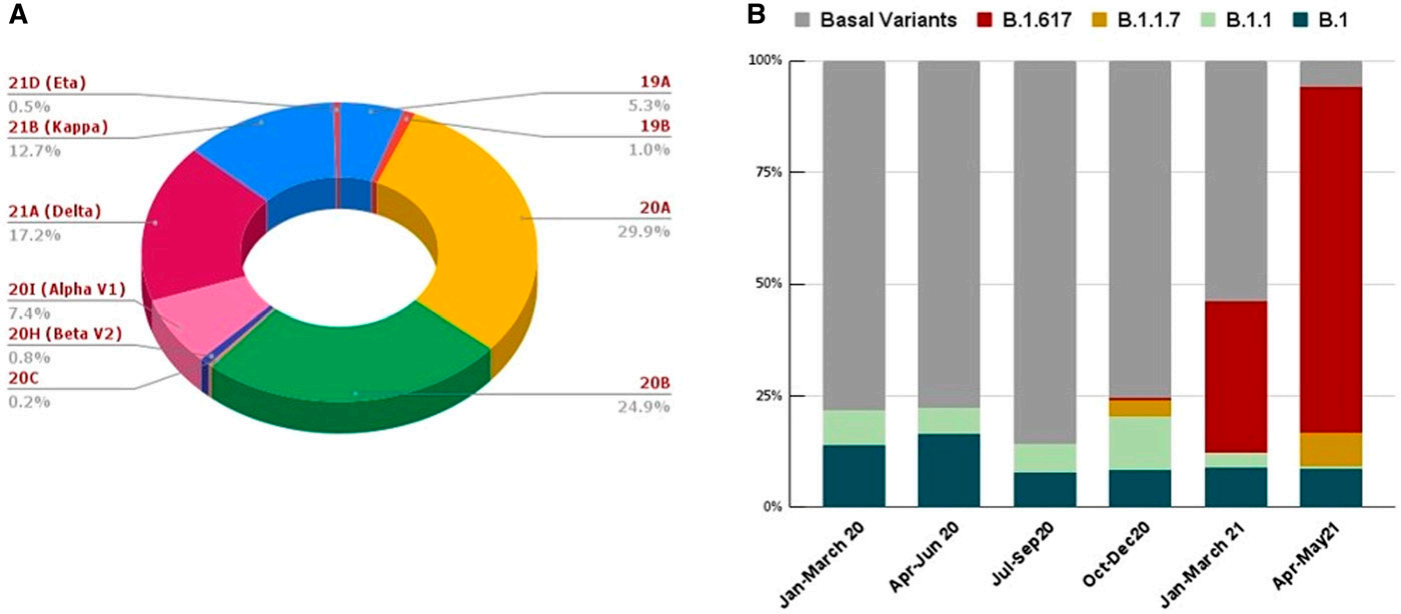
(**A**) Overall distribution of severe acute respiratory syndrome coronavirus 2 (SARS-COV-2) clades according to Nextstrain classification. (**B**) Temporal distributions of the top five variants (represented in cumulative percentage) of SARS-COV-2 genomes. This figure appears in color at www.ajtmh.org.

To understand the evolution of SARS-COV-2 in India, phylogenetic analysis was performed on the available genome dataset. Phylogenetic analysis shows that the reported SARS-COV-2 genomes in India were dispersed across the evolutionary tree of SARS-COV-2 (Figure 1). From this analysis, it is apparent that there are six major clades with varying degrees of percentages of Indian SARS-COV-2 cases: 19A (5.3%), 20A (29.9%), 20B (24.9%), 20I-Alpha, V1 (7.4%), and 21A-Delta (17.2%), and 21B-Kappa (12.7%). Other clades found in Indian SARS-COV-2 genomes include 19B, 20C, 20H, and 21D. The overall distribution of SARS-COV-2 lineages circulating in India during the study period is represented in Figure 2; corresponding numbers observed in different quarters are presented in Table 1.

**Table 1:** Correspondence analysis indicating the change in the dominance of severe acute respiratory syndrome coronavirus 2 (SARS-COV-2) variants over the time period

Period	Quarter code	Basal variants	B.1	B.1.1	B.1.1.7	B.1.617	Total
Jan.–March 2020	Q1	193	40	20	0	0	253
April–June 2020	Q2	2,375	516	190	0	0	3,081
July–Sept. 2020	Q3	2,029	183	164	0	0	2,376
Oct.–Dec. 2020	Q4	1,525	142	243	75	11	1,996
Jan.–March 2021	Q5	1,823	427	148	718	1,600	4,716
April–May 2021*	Q6*	192	276	21	243	2,586	3,318
Total of SARS-COV-2 genomes		8,137	1,584	786	1,036	4,197	15,740

Chi-square value = 9,600.421; significant at 0.0001.

* Q6 is an incomplete quarter as it covers data from only 2 months.

In contrast, the PANGO lineage scheme showed the existence of four major lineages—“A” (128/14526), “B” (14359), “C” (8), “L” (1), “N” (3), and “P” (4)—of SARS-COV-2; lineage B was the dominant lineage and was further divided into several sublineages. Among the several sublineages of B; the newly emerged variants such as B.1.617.1 and B.1.617.2 lineage belonging to the next strain clade 21A-Delta and 21B-Kappa constitute 17.2% and 12.7%, respectively, of the total SARS-COV-2 sequences reported from India. The observed genetic diversity also evinces the multiple introductions of SARS-COV-2 from different sources and the emergence of new lineages within the country due to the accumulation of unique mutations in the viral genome giving rise to the diversity in the virus lineages. Among the different components of SARS-COV-2, spike protein has been identified as a hotspot for mutations that has clinical implications in terms of virulence, transmissibility, and host immune evasion. The characteristic spike protein mutations in the emerging SARS-COV-2 variants and its potential implications in the clinical management of COVID-19 have been reviewed extensively by several investigators.^13^^,^^18^^,^^20^ This study highlights considerable phylogenetic divergence among SARS-COV-2 in India, which may play a pivotal role in the transmissibility, virulence, and ability to invade new hosts of the virus, as well as disease severity.

The first genome sequence of B.1.617 emerged from the western part of India in October 2020 and was characterized by key mutations in the spike protein such as D111D, G142D, L452R, E484Q, D614G, and P681R.^22^ The B.1.617 and its sublineages reported from India are popularly known as “double mutant” because their genome carries two critical amino acid substitutions, such as L452R and E484Q, in the receptor binding domain (RBD) of the S glycoprotein apart from other nonsynonymous mutations. Another unique mutation, P681R, located in the S1–S2 furin cleavage site of spike protein but outside the RBD region, is also common to all the newly emerged lineages of B.1.617.^22^ It was found from past investigations that strains with substitutions L452R and E484Q in spike protein confer a stronger affinity for the angiotensin-converting enzyme-2 receptor of the host for the viral attachment and diminished recognition ability of the immune system.^8^ Considering the possible contribution of specific mutations to increased transmissibility, severity, and immune escape, the WHO and other international health authorities have categorized the B.1.617.2 (21A-Delta) as a VOC.^8^^,^^14^ Among the observed lineages, B.1.617 and its sublineages (21A-Delta and 21B-Kappa) are the two notable clades that dominated nearly 78% of the total SARS-COV-2 genomes reported to GISAID from India during April and May 2021. Although the B.1.617.2 variant of SARS-COV-2 originated in India, it has disseminated to at least 80 counties, including the United States, where the Delta variant was detected in 49 states in a short time span. Henceforth, B.1.617 and its sublineages were designated as B.1.617 without subdividing its sister lineages to simplify the downstream statistical calculation. The top distinct lineages (e.g., B.1, B.1.1, B.1.1.7, and B.1.617) and basal sequences reported during the study period are depicted in Figure 2.

To understand the abundance of the top lineages of SARS-COV-2 with time, correspondence analysis was performed on the available genome dataset (Table 1, Supplemental Figure 2). From the analysis, it was observed that during the first three quarters, a predominant proportion of the infections corresponded to the basal sequences and a minor number from B.1 and B.1.1. Two SARS-COV-2 VOCs, B.1.1.7 and B.1.617, emerged in the fourth quarter, and a few cases corresponded to them as well, although the majority were still associated with SARS-COV-2 basal sequences of non-VOCs. The newly emerged B.1.617 lineage was first identified in India in September 2020 and was designated as a VOC on December 14, 2020. From the correspondence analysis, a transition was evident in the fifth quarter (January–March 2021), as B.1.617 and/or its sublineages emerged as key contributors to the majority of COVID-19 cases reported during the sixth quarter (April–May 2021). Supplemental Figure 2 shows that the fifth quarter diverged from the basal sequences of SARS-COV-2, with cases more often related to B.1.617 and/or its sublineages; this contributed to the equidistribution of infected cases.

Nearly 78% of the SARS-COV-2 genomes reported from India during the sixth quarter (April–May 2021) belonged to B.1.617 and/or its sublineages. This implies that during this period, COVID-19 infections were strongly related to B.1.617 and/or its sublineages and moved away from the basal SARS-COV-2 lineages reported during the early phase of the COVID-19 pandemic in India. Chi-square test shows that this divergence is found statistically significant at a 5% level. B.1.617 and its sublineages dominated during the peak of the second wave (sixth quarter; April–May, 2021) characterized by rising infections, hospitalizations and deaths. In a short period, the B.1.617 (Delta) variant of SARS-COV-2 emerged as a major strain across Indian states and was transmitted to more than 40 countries, crippling health systems globally. Molecular epidemiological studies and experimental evidence from different sources suggest that this variant presents the dual challenge of reduced vaccine efficacy and increased transmissibility beyond the previously circulating lineages of SARS-COV-2. Bolze et al. showed that until March 2021, the majority of COVID-19 cases in the United States were cause by B.1.1.7; later, it was displaced by the newly emerged B.1.617.2, with higher growth rates comparable to B.1.1.7.^23^ According to preliminary analysis of COVID-19 cases, increased dominance of B.1.617.2 over the existing B.1.1.7 variant was observed in the Canadian province of Ontario.^24^ The surge in COVID-19 cases due to variants of B.1.617 and/or its sub lineages in different geographic areas provides a clue to the selective advantage of this variant. Several unique mutations reported in the genome of B.1.617 and/or its sublineages might have contributed to the selective advantage of the virus to become dominant during the explosive growth of COVID-19 cases in the second wave. A recent report observed that in several COVID-19–affected countries, new variants of SARS-COV-2 are arguably responsible for reinfections after either natural infection or vaccination.^8^ A study by Mukherjee et al. has provided evidence on COVID-19 reinfections in India and the potential threat to vaccinated individuals.^25^

### Age distribution pattern of deceased individuals infected with the top five SARS-COV-2 variants.

Exploratory studies on the genomic and pathogenic properties of newly emerged SARS-COV-2 variants are progressing in tandem with the paucity of information on epidemiological characteristics, clinical outcomes, and mortality, particularly among patients infected with these newly emerged variants. This study examined the age distribution pattern of deceased individuals infected with the top five variants of SARS-COV-2 for the available data in India, and it is noted that the age of the patients deceased from COVID-19 is not widely dispersed, but rather skewed at the higher age level (Supplemental Figure 3A and B) irrespective of the type of SARS-COV-2 variants. The Kruskal-Wallis test (test statistic = 2.998; *P* = 0.558) indicates that there is no statistically significant difference between the SARS-COV-2 variants based on age of the deceased patients.

Global estimates of the COVID-19 burden show a consistent and distinct pattern of an age-based exponential increase in fatality rate across nations, implying that the age of infected persons, along with sex and comorbidities, is a major predictor of mortality.^26^^,^^27^

Age of the deceased is high across all variants, implying that elderly individuals infected by any variant of COVID-19 are at the high risk of mortality (refer to the mean values). Furthermore, the average age of the deceased belonging to any variant is older than 55 years; this indicates that the health status age of an individual are associated, irrespective of patient age. Also, the deceased were older, and their chances of dying from the infection was greater compared with other age groups. This finding offers an opportunity to build a model measuring the impact of age on health status (deceased or nondeceased). However, a wide and statistically significant difference could be observed for recovered patients infected by the different VOC based on their age (Kruskal-Wallis test: test statistic = 14.723; *P* = 0.005). Mean recovery age is lowest for B.1.617 (40 years) and highest for B.1.1.7 (54 years). It implies that COVID-19 is more fatal among people infected by the Delta variant than the B.1.1.7 variant. It is alarming that the Delta variant has reduced the average age of recovery. Furthermore, pairwise comparison tests were performed, and the results showed a statistically significant difference in mortality occurring from the infection of variant B.1 and variant B.1.167 (Supplemental Table 1). It is noteworthy that the deaths from variant B.1 also appear to be different from basal SARS-COV-2 sequences at a 10% level of significance. A retrospective analysis of patients with COVID-19 in England showed that adults aged > 30 years are at higher risk for B.1.1.7 variant than wildtype SARS-COV-2.^8^ This study also analyzed whether there was any gender-based difference in the mortality across the different COVID-19 variants.

It was hypothesized that there is no significant difference between male and female patients with respect to mortality, and this was tested with the chi-square test (Supplemental Table 2). The result does not reject the null hypothesis, implying that the risk of mortality from COVID-19 is not dissimilar between male and female patients, and this observation corroborates the findings of Peckham et al., obtained from a large-scale meta-analysis of 3,111,714 global COVID-19 cases in which males and females were at equivalent risk of infection.^29^

### Estimating the risk of mortality associated with SARS-COV-2 variants.

In an interim analysis, the Indian Council of Medical Research demonstrated that the pathogenic potential of SARS- COV-2 VOCs dominated during the second wave of COVID-19 (January–June 2021) in India, which profoundly influenced the transmissibility of the virus and increased the risk of hospitalization and mortality. Other major concerns associated with new variants of SARS-COV-2 were their potential role in reinfections either after natural infection or vaccination, immune escape, and possible failure of quantitative RT-qPCR. Considerable evidence exists on immune evasion of natural and vaccine-induced antibodies by these new variants of SARS-COV-2.

This study evaluates whether the proportional change of SARS-COV-2 variant spread during the COVID-19 epidemic in India has any association with mortality using a multivariable logistic regression model. To the best of our knowledge, the association of the dominant SARS-COV-2 variant, including newly emerged VOC B.1.617 reported in India to the COVID-19 mortality, has not been investigated so far in this broader context. Hence, the present study enables us to fill the void by providing substantial insight. Apart from estimating the vulnerable variant type, the study also measures whether any difference exists in the chance of mortality of infected individuals belonging to different age groups or by sex (Table 2). The coefficient values reported in Table 3 estimate the size of log odds of demised COVID-19–infected individuals against recovery. They are estimated by deriving the exponent of the coefficients derived from estimating Equation (1), which are also presented in Table 3.

**Table 2:** Predicting the risk of mortality associated with different severe acute respiratory syndrome coronavirus 2 variants in India

Attribute	Regressor	Estimate	SE	z-value	Pr(>|z|)
	(Intercept)	−3.49569	0.33878	−10.319	< 2e-16***
Age, years	45–65	2.33262	0.31716	7.355	1.91e-13***
	> 65	3.14913	0.34095	9.236	< 2e-16***
Gender	Male	−0.04824	0.20972	−0.23	0.8181
Variants	B.1	−0.65619	0.29514	−2.223	0.0262**
	B.1.1	−1.15945	1.0695	−1.084	0.2783
	B.1.1.7	−0.27807	0.84761	−0.328	0.7429
	B.1.617	1.76113	0.2888	6.098	1.07e-09***

Significance: ***0.01; **0.05; *0.10.

**Table 3:** Exponential of coefficient values measuring the chances of death of individuals infected with the variants of concern

Regressors	Intercept	45–65 years	≥ 65 years	Male	B.1	B.1.	B.1.1.7	B.1.617
Exponential of coefficients	0.03	10.3	23.31	0.95	0.51	0.31	0.75	5.81

Base category: individuals infected with basal variant, female, and aged < 45 years.

This analysis demonstrates that the coefficient value of the intercept term is 0.030 (Table 3) and implies that the probability of mortality against recovery of COVID-19 cases in the base category (females aged ≤ 45 years infected with basal SARS-COV-2) is 0.03, and the probability of recovery is 0.97.

However, the odds of mortality of the individuals infected with basal SARS-COV-2 increase if the infected patient is in the older age group. The chances of mortality of infected individuals in the age group of 45 to 65 years appear to be 10 times higher than young individuals (≤ 45 years), and it is 23 times higher among older people (≥ 65 years) individuals than young though they are all infected with basal SARS-COV-2. Hence, the lower risk of mortality among young and the high risk of mortality among old is reinforced. Regarding individuals’ sex, the odds ratio of males is 0.95, which is almost equal to 1. It implies that the chances of mortality of males are more or less equal to females who are infected with basal SARS-COV-2. It thus confirms the result of the chi-square test indicating that there is no significant difference in the odds of mortality between male and female patients.

Furthermore, this study has identified changes in mortality from SARS-COV-2 with the mutations taking place in the VOCs. If an individual is infected with a B.1 variant, the chance of death is less than an individual infected with basal SARS-COV-2 virus. Similarly, the probability of death of individuals infected with SARS-COV-2 B.1.1 or B.1.1.7 is less than individuals infected with basal SARS-COV-2 virus. The SARS-COV-2 variant B.1.1.7 was first detected in the United Kingdom in December 2020 and later transmitted to 110 countries, including India. Recent hospital- and community-based epidemiological studies show that patients infected with B.1.1.7 are at increased risk of critical care admission with a substantial increase in mortality compared with previously circulating SARS-COV-2 variants.^26^^,^^30^ The systematic analysis of severe outcomes associated with SARS-COV-2 B.1.1.7 in England shows that admission for critical care was 2 times higher among individuals infected by the Delta variant compared with the basal SARS-COV-2.^31^ However, this finding contrasts with previous studies and did not confer an increased risk of mortality in patients infected with B.1.1.7 compared with the basal SARS-COV-2, which could be due to the smaller dataset reported from India to GISAID with clinical outcomes and insufficient genomic surveillance. According to the recent report published in the *British Medical Journal*, there is a paucity of genomic and epidemiological data on the different SARS-COV-2 variants circulating in different regions of India, which hampers understanding of the complex epidemiology of COVID-19 cases and underscores the need for formulating specific strategies to curb the spread and severity of the virus.^32^ The striking observation from the present analysis is the association of B.1.167 with an increased risk of death in relation to the basal sequences. The coefficient value of the variant B.1.167 indicates that the chance of death of individuals infected with variant B.1.167 is 5.8 times greater than the individuals infected with basal SARS-COV-2. This implies that the risk of mortality is high in cases with variant B.1.167 compared with basal SARS-COV-2.

The model was tested for good fit using Hosmer-Lemeshow test, and it appears that the logistic regression model is a good fit for the given data and results (χ^2^ = 2.85, *df* = 8, *P* value = 0.943). Further, the study also examined the odds ratio of all possible combinations of the model (Supplemental Table 3). From the odds ratio of 29 possible combinations of different VOCs, age group, and gender of the deceased, it is evident that the chances of dying if infected by SARS-COV-2 B.1.617 are many times higher than they are with other variants; and chance of death increases further if infected individuals are in the older age categories (45–64 years and > 65 years) irrespective of gender. The increased infectivity and severity of newly emerged variants in India has thus been reported. These estimates reflect a substantial increase in the risk of mortality among older age groups due to India’s newly emerged SARS-COV-2 variant. The findings of this study support the laboratory findings on the implications of newly emerged variants on the pathogenesis and clinical outcome. In toto, this study reveals an increased risk of mortality associated with the B.1.617 lineage.

### Study Limitations.

In this study, which analyzed a COVID-19 dataset available from a public database, we were faced with certain data constraints, particularly due to the nature of the SARS-COV-2 genomic and associated epidemiological data of such patients. As a precautionary measure, we took utmost care in data curation, to minimize the confounding effects that otherwise would have influenced the outcome of this study. Considering the skewed nature of the genome and the associated epidemiological data of the COVID-19 cases available in the public database, we reiterate the need for a robust and unified algorithm for sample prioritization criteria, collection of patient metadata, and selection of appropriate sequencing methods that would support global efforts to design more effective healthcare strategies to combat COVID-19.

## CONCLUSION

This study analyzed the characteristics of SARS-COV-2 genomes reported from India and examined whether the chances of mortality change with the change in the proportion of SARS-COV-2 variants spread during the COVID-19 epidemic using multivariable logistic regression models. SARS-COV-2 genomes are dispersed across the evolutionary tree. During the second wave of the pandemic in India, a large number of COVID-19 infections were associated with the newly emerged B.1.617 variant and/or its sublineages, with an increased risk of hospitalization and death. This study shows that the mean recovery age for B.1.617 (40 years) is the lowest, whereas it is highest for B.1.1.7 (54 years). This implies that the average age of patients who recovered from COVID-19 infection is much lower among those infected by Delta variant (B.1.617) and its sublineages than those who contracted the B.1.1.7 variant. Furthermore, the study also revealed that a significant increase in the chances of COVID-19 mortality was associated with patients infected by the Delta variant (B.1.617) and/or its sublineages compared with other variants. This risk is more pronounced among those infected by the Delta variant or who are aged > 45 years, regardless of sex. Drawing from the results, the study suggests that vaccine-breakthrough cases by highly transmissible viral variants can potentially disrupt the management of the COVID-19 pandemic. It is thus necessary to continuously generate viral genomic sequences from positive samples to identifying potential vaccine-breakthrough mutations. Even though this research is preliminary, the outcome of this study, along with observations on increased infections, hospitalization, and mortality and a few reports of vaccine-breakthrough cases, highlights the importance of clinically characterizing B.1.617 and/or its sublineages. Another key concern stemming from the prevailing circumstances is the insufficiency of evidence to determine the effect of B.1.617 and/or its sublineages on vaccinated individuals, how long vaccine protection against the variant lasts, and whether breakthrough infections are more severe. Considering the transmissibility of the B.1.617 and/or its sublineages and its potential impact on public health, developing a socioeconomic response framework to protect the needs and rights of the most vulnerable people living under the risk of the pandemic should also be a focus of research. With changes in risk factors for COVID-19 due to emerging variants, strategic measures and policy approaches can be realigned for effective management of the pandemic. Future research should also focus on systematic collection and reporting of detailed, robust, patient-level data of COVID-19 cases coupled with stringent genomics surveillance to investigate the impact of newly emerged variants by building robust models to assess the epidemiological burden on public health. Such an integrated approach of clinical-genomic models for COVID-19 would benefit the stakeholders in developing appropriate clinical and public health strategies that not only contain the spread of the epidemic but also prevent the emergence of COVID-19 waves in the future.

## Supplemental tables


Supplemental materials
