# Supplementary material for: The Dominance of Severe Acute Respiratory Syndrome Coronavirus 2 B.1.617 and Its Sublineages and Associations with Mortality during the COVID-19 Pandemic in India between 2020 and 2021
Source: Am J Trop Med Hyg. 2021 Nov 17;106(1):142–9. doi: 10.4269/ajtmh.21-0812 (PMC8733510; doi:10.4269/ajtmh.21-0812)
Supplement: Supplementary file 1 [file tpmd210812.SD1.pdf]

Legends for supplementary figures

Fig S1. Distribution of most frequently found non-synonymous mutations in the Indian SARS-COV-2

Fig. S2. Correspondence analysis showing the dominance of different SARS-COV-2 variants in relation to time

[*Note: Var-1 = Basal sequences; Var-2 = B.1; Var-3= B.1.1; Var-4=B.1.1.7 and Var-5= B.1.617 and its sub lineages; Q1=Jan-March, 2020; Q2=April-June, 2020' Q3= July-Sept, 2020; Q4= Oct-Dec, 2020; Q5= Jan-March, 2021; Q6=April-May, 2021*]

Fig. S3. Age distribution of (A) recovered and (B) deceased individuals from different SARS-COV-2 variants

Supplementary Tables

Table S1. Age specific differences in the recovery and mortality of patients infected with different SARS-COV-2 variants

Variant Type	Clinical Outcome					
	Age of Recovered			Age of Deceased		
	Mean	Median	Standard Deviation	Mean	Median	Standard Deviation
SARS-COV-2 basal variants (N=571)	43	43	17	62	62	12
B.1 (N=147)	48	49	17	61	64	16
B.1.1 (N=14)	46	53	19	57	57	
B.1.1.7 (N=11)	54	55	16	66	66	29
B.1.617 and its sub lineages (N=92)	40	36	16	57	58	14
Kruskal-Wallis test	Test Statistic=14.723 p-value=0.005			Test Statistic=2.998 p-value=0.558		
Pairwise Comparisons	Significant difference between Variants 2 and 5. Test Statistic=3.022, p-value=0.025			NA		

Table S2. Gender-based difference in the risk of COVID-19 mortality

	Value	df	Asymptotic Significance (2-sided)	Exact Sig. (2-sided)	Exact Sig. (1-sided)
Pearson Chi-Square	2.074a	1	0.15		
Continuity Correction	1.812	1	0.178		
Likelihood Ratio	2.037	1	0.153		
Fisher's Exact Test				0.158	0.09

Table S3. Odds ratio of all possible combinations indicating the chances of mortality against recovery from COVID-19 infection

Combinations	Odds Ratio
Base Category (Basal Variant, Female, Age_<=44) or Intercept	0.030
Basal Variant, Female, Age_>=45_<=64	10.335
Basal Variant, Female, Age_>=65	23.346
Basal Variant, Male, Age_<=44	0.983
Basal Variant, Male, Age_>=45_<=64	11.288
Basal Variant, Male, Age_>=65	24.299
B.1, Female, Age_<=44	0.549
B.1, Female, Age_>=45_<=64	10.854
B.1, Female, Age_>=65	23.865
B.1, Male, Age_<=44	1.502
B.1, Male, Age_>=45_<=64	11.807
B.1, Male, Age_>=65	24.818
B.1.1, Female, Age_<=44	0.344
B.1.1, Female, Age_>=45_<=64	10.649
B.1.1, Female, Age_>=65	23.660
B.1.1, Male, Age_<=44	1.297
B.1.1, Male, Age_>=45_<=64	11.602
B.1.1, Male, Age_>=65	24.613
B.1.1.7, Female, Age_<=44	0.788
B.1.1.7, Female, Age_>=45_<=64	11.092
B.1.1.7, Female, Age_>=65	24.103
B.1.1.7, Male, Age_<=44	1.740
B.1.1.7, Male, Age_>=45_<=64	12.045
B.1.1.7, Male, Age_>=65	25.056
B.1.617, Female, Age_<=44	5.849
B.1.617, Female, Age_>=45_<=64	16.154
B.1.617, Female, Age_>=65	29.165
B.1.617, Male, Age_<=44	6.802
B.1.617, Male, Age_>=45_<=64	17.107
B.1.617, Male, Age_>=65	30.118

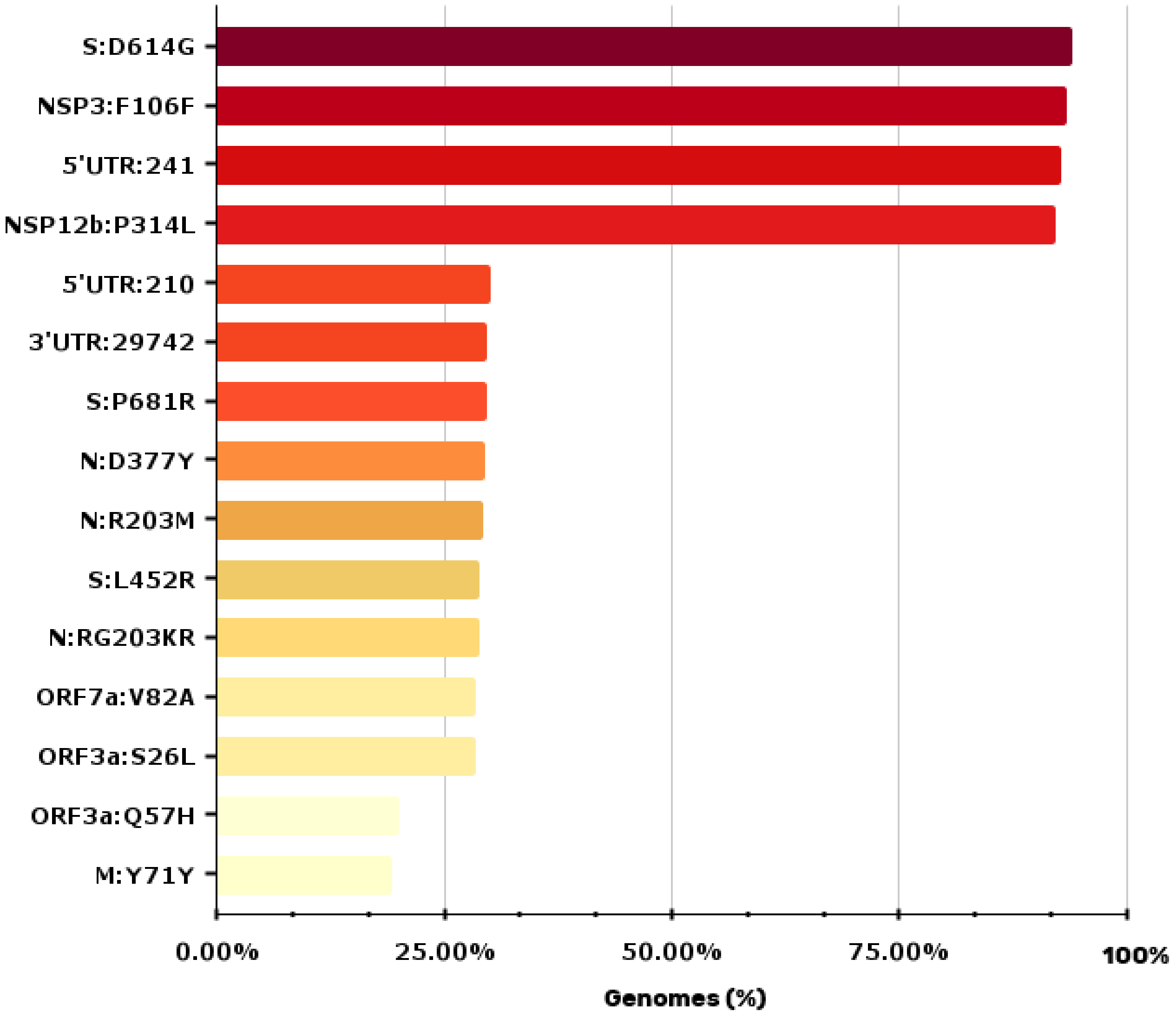
S:D614G
NSP3:F106F
5'UTR:241
NSP12b:P314L
5'UTR:210
3'UTR:29742
S:P681R
N:D377Y
N:R203M
S:L452R
N:RG203KR
ORF7a:V82A
ORF3a:S26L
ORF3a:Q57H
M:Y71Y
0.00%
25.00%
50.00%
75.00%
100%
Genomes (%)

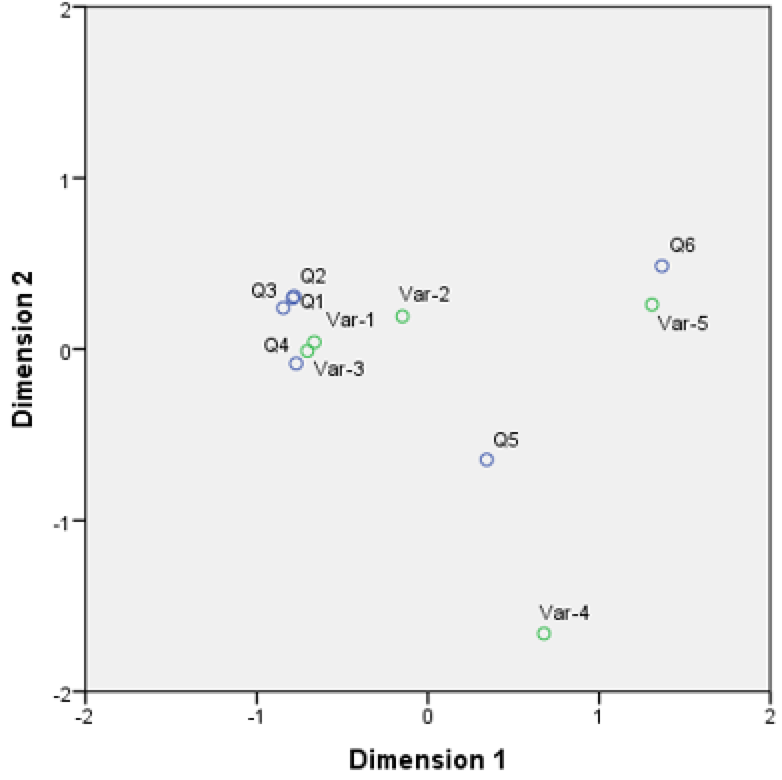

Dimension 2
Dimension 1
2
1
0
-1
-2
-2
-1
0
1
2
Q1
Q2
Q3
Q4
Q5
Q6
Var-1
Var-2
Var-3
Var-4
Var-5

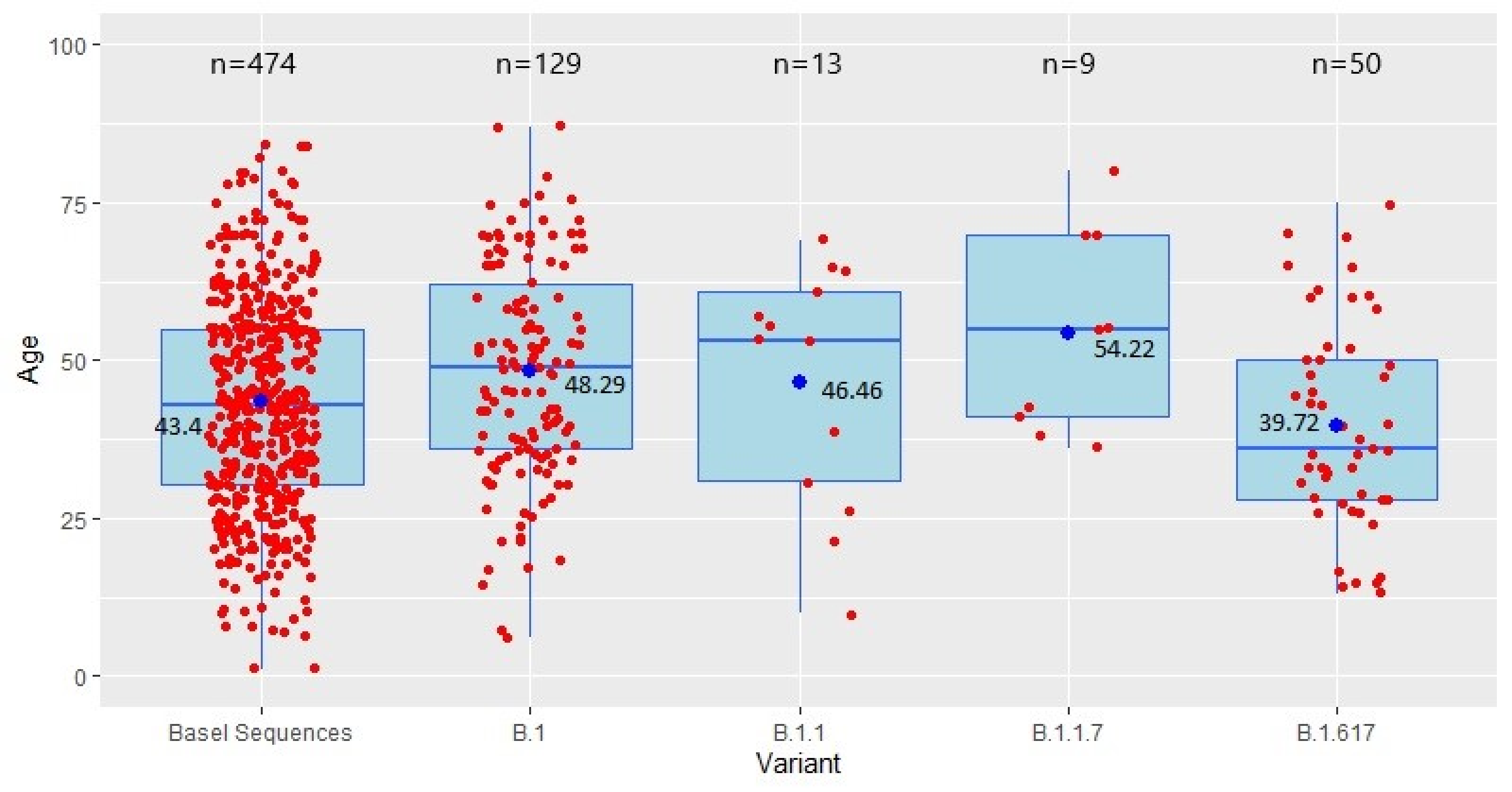
n=474
n=129
n=13
n=9
n=50
100
75
50
25
0
Age
43.4
48.29
46.46
54.22
39.72
Basel Sequences
B.1
B.1.1
B.1.1.7
B.1.617
Variant

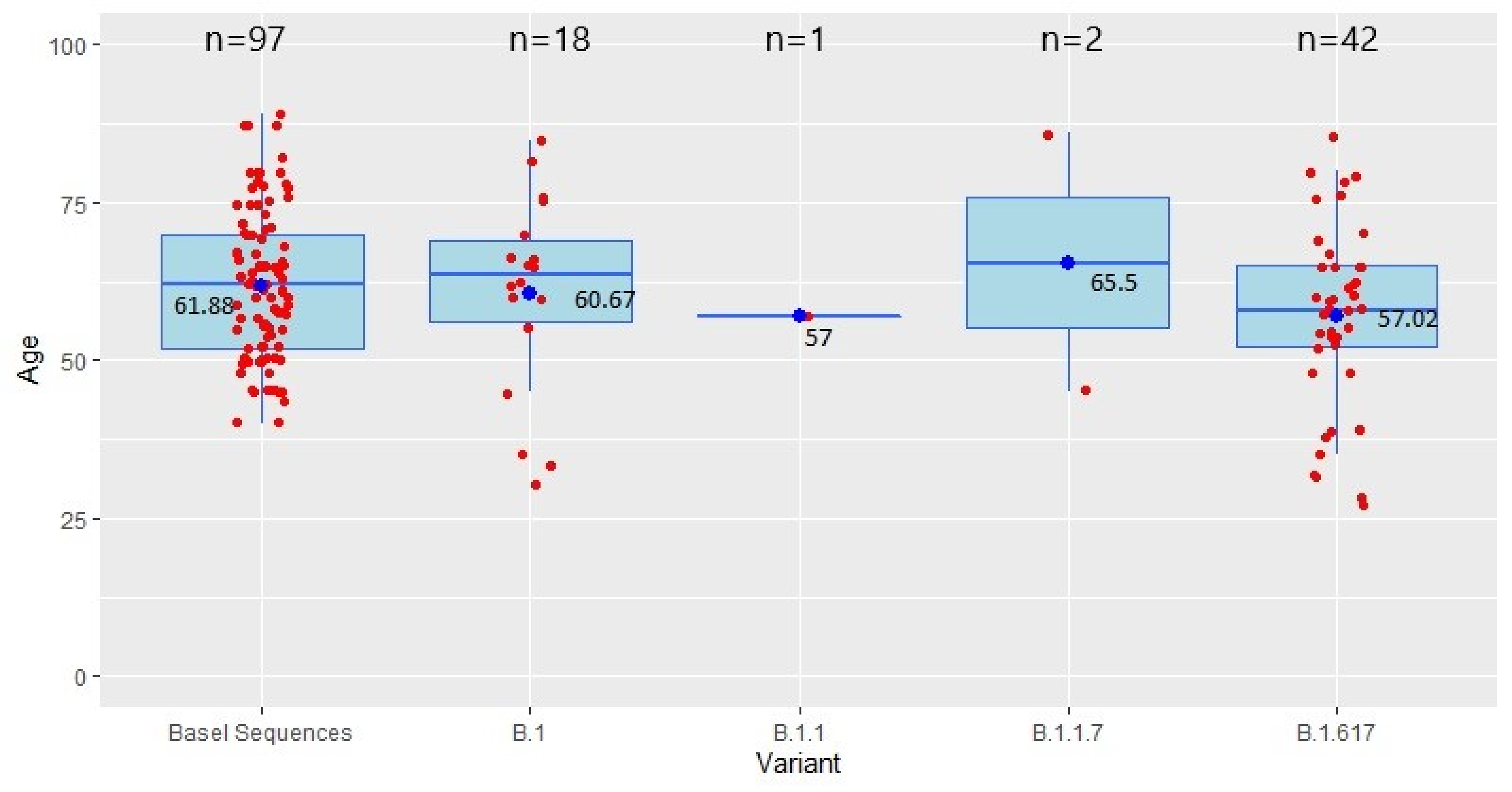

n=97
n=18
n=1
n=2
n=42
100
75
50
25
0
Age
61.88
60.67
57
65.5
57.02
Basel Sequences
B.1
B.1.1
B.1.1.7
B.1.617
Variant
